# Gut Metabolome in Companion Animal Nutrition—Linking Diets to Health

**DOI:** 10.3390/ani15050651

**Published:** 2025-02-24

**Authors:** Yang Lyu, Junning Pu, Baichuan Deng, Caimei Wu

**Affiliations:** 1 Key Laboratory of Animal Disease-Resistance Nutrition, Sichuan Province, Ministry of Agriculture and Rural Affairs, Ministry of Education, Animal Nutrition Institute, Sichuan Agricultural University, Chengdu 611130, China; yang.lyu@sicau.edu.cn (Y.L.);; 2 Guangdong Provincial Key Laboratory of Animal Nutrition Control, College of Animal Science, South China Agricultural University, Guangzhou 510642, China

**Keywords:** pet food, metabolomics, gut health, cats, dogs

## Abstract

Tailoring diet to support a healthy gut microbiome is key for animal well-being. The gut metabolome, including all small molecules in the gut, is central to diet–microbiome–health interactions. While comprehension of the gut metabolome in companion animal nutrition is emerging, a substantial number of studies have been undertaken to elucidate the metabolomic shifts and identify specific marker metabolites influenced by diverse dietary interventions. By employing various metabolomic approaches, researchers have extensively documented the effects of different diet types, nutrient compositions, and dietary supplements on the gut metabolome in dogs and cats. Despite these advancements, there remains several notable limitations, including a lack of integrated microbiome analysis, incomplete understanding of specific marker metabolites, and an over-reliance on extrapolating findings from human studies. Therefore, this review aims to summarize the current understanding of the canine and feline gut metabolome, while exploring future possibilities and challenges for the field.

## Simple Summary

Customizing dietary strategies for a healthy gut microbiome is paramount to animal well-being. The gut metabolome plays a pivotal role in mediating diet–microbiome–health interactions. Companion animal gut metabolome knowledge is an emergent subject, with many studies identifying diet-driven shifts. However, research lacks integrated microbiome analysis, has incomplete marker understanding, and over-relies on human data. This review summarises the canine and feline gut metabolome and explores future challenges.

## 1. Introduction

From a chemical standpoint, conventional canine and feline diets consist of roughly 8000 distinct compounds, encompassing dietary fibres, polyphenols, and other constituents [1,2]. Some of these compounds are not digestible by the host’s endogenous enzymes, and remain unaltered as they pass through the gastrointestinal (GI) tract [3]. Within the gut, these undigested components are then subject to breakdown by the resident microbiota [4]. This process generates a diverse array of downstream metabolites, collectively termed the gut metabolome [3,4].

It has been suggested that specific profiles of the gut metabolome, as well as the abundance of particular metabolites, may offer pivotal information on the metabolic effects of the microbiome on host health [5]. From a nutritional perspective, in addition to the consequences of dietary changes or adaptions, gut metabolomic alterations have been associated with the development and progression of various metabolic diseases in dogs and cats, including diabetes [6], obesity [7], and (chronic) liver [8], kidney [9], and gut disease [10]. More specifically, elevated concentrations of short-chain fatty acids (SCFAs) have been correlated with declined adiposity and enhanced glucose metabolism in canine and feline studies [7,11]. Conversely, increased branched-chain amino acid (BCAA) levels have been linked to insulin resistance and an elevated susceptibility to obesity in dogs [12].

Nevertheless, in-depth investigation of the gut metabolome is still relatively nascent, with the majority of current research focusing on studying the metabolome in the context of GI and kidney diseases [13,14]. Research on the gut metabolome in healthy dogs and cats, particularly regarding canine and feline nutrition, remains limited [15,16].

This review focuses on the gut metabolome within the context of pet nutrition research, summarises current knowledge regarding the influence of dietary interventions on the gut metabolome of healthy dogs and cats, encompassing aspects including the composition of the gut metabolome, methodologies for its characterisation, the impact of varying nutrient profiles, diet types, and dietary supplementation. Finally, the review also acknowledges limitations in current understanding and outlines potential areas for future research.

## 2. Materials and Methods

All cited sources in this review were retrieved from the following publicly accessible databases: Google Scholar (https://scholar.google.com/ (accessed on 1 January 2024)), PubMed (https://pubmed.ncbi.nlm.nih.gov/ (accessed on 1 January 2024)), ScienceDirect (https://www.sciencedirect.com/ (accessed on 1 January 2024)), Web of Science (https://www.webofscience.com/ (accessed on 30 August 2024)), Embase (https://www.embase.com/ (accessed on 30 August 2024)), and Scopus (https://www.scopus.com/ (accessed on 30 August 2024)). The search was performed using different combinations of the following keywords: gut/gastrointestinal/faecal, metabolism/metabolome/metabolomics, dogs/cats/pets, and *Canis familiaris*/*Felis catus*, with a time frame from January 2000 to December 2024. Only articles with titles and abstracts written in English were included.

## 3. Gut Metabolome and Key Metabolites in Dogs and Cats

Similarly to humans and other animals, the primary contributors to the gut metabolic pool in dogs and cats are proteins, carbohydrates, and fats [17]. These macronutrients undergo breakdown during digestion, resulting in the formation of various metabolites, including gases (e.g., CO_2_, CH_4_), organic acids (e.g., lactate, pyruvate), biogenic amines (e.g., histamine, serotonin), SCFAs (e.g., butyrate, acetate), branched-chain fatty acids (BCFAs, e.g., iso-butyrate, iso-valerate), and BCAAs (e.g., iso-leucine, valine) (Table 1). These metabolites play critical roles in maintaining host physiological processes [18]. The gut also comprises a diverse range of metabolites derived from bacterial cell wall components, polyamines, volatile organic compounds, plant secondary metabolites, and dietary choline [19]. These metabolites are highly dynamic and undergo various processes, including absorption, distribution, and/or excretion from the body [7,11]. This dynamic nature underscores the intricate complexity of gut metabolism and its profound influence on overall health and well-being [1,2,3,4].

The vast majority of metabolomic studies in cats mainly used serum or plasma samples, with very few studies exploring the gut or faecal metabolome [9,20,21,22]. To date, Honneffer et al. [23] have provided the only currently available study reporting the metabolomic profile along the healthy canine GI tract. In this study, a total of 530 metabolites were detected in the duodenal, ileal, colonic, and rectal contents of healthy dogs, of which levels of 141 metabolites were significantly different among the four intestinal sections. Notable findings included that the relative concentrations of multiple amino acids (e.g., valine, leucine, alanine) gradually declined along the four intestinal sections, whilst several phenol-containing carboxylic acid compounds (e.g., 3-phenyllactic acid, 4-hydroxyphenylacetic acid) gradually increased. In addition, it was found that the measurable levels of pyruvate were highest in the ileum [23]. These findings can be viewed as an early study, providing a foundation for more research.

In general, from the perspective of production volume and health implications, the dominant metabolites in the canine and feline colon are primarily SCFAs, secondary bile acids, and BCFAs and indole derivatives, which arise from the fermentation of carbohydrates, fats, and proteins, respectively.

### 3.1. SCFAs

Short-chain fatty acids (SCFAs), the primary bacterial metabolites produced through the fermentation of dietary fibre and resistant starches by specific anaerobic bacteria (e.g., *Roseburia*, *Bacteroides*) within the colon, are crucial for animal health [24,25]. These SCFAs exhibit numerous health benefits in humans and companion animals alike, including anti-inflammation, immunomodulation, weight management, diabetes management, and cancer prevention, as well as protective effects on organs such as the heart and liver [24,25]. Moreover, SCFAs can reduce the intestinal pH and prevent the absorption of harmful metabolites such as ammonia [26]. Notably, unlike most omnivorous mammals that produce SCFAs from the fermentation of dietary fibre and resistant starches, dogs and cats, as carnivores, have more abundant protein-fermenting bacteria (e.g., *Clostridium perfringens*) in their colon that are capable of producing SCFAs [27,28]. Additionally, while the direct contribution of protein to SCFA production is less important than fibre [27], it can indirectly influence the process through changes in gut microbiome composition, transit time, and utilisation of specific amino acids [29,30]. This fascinating field remains unexplored in canine and feline nutrition research. Further investigation is needed to clarify the complex interactions among proteins, gut bacteria, and SCFA production.

Acetate, propionate, and butyrate, the three major SCFAs, have been extensively studied for their diverse biological activities [31]. Acetate can be utilised by muscle tissue as a source of energy [32]. Propionate is absorbed by the liver from the portal blood and transformed into succinyl-coenzyme A, which is involved in energy production, the citric acid cycle, ketone metabolism, and haem synthesis [33]. Butyrate is oxidised by the colon and serves as the preferred energy substrate for colonocytes [34].

The production of SCFAs has been frequently reported on in both canine and feline nutritional research across various dietary interventions and supplementation strategies [27,28]. Plant-derived oligosaccharides (e.g., fructo-, galacto-, manno-, and xylo-oligosaccharides (FOSs, GOSs, MOSs, XOSs)) and various types of fermentable carbohydrates (e.g., pectin, inulin, and resistant starch) sourced from multiple food ingredients have consistently demonstrated significant increases in SCFA production in numerous recent dog and cat studies [35,36,37,38,39,40]. Specifically, studies have demonstrated elevated levels of butyrate, acetate, and/or valerate in adult canines when their diets were supplemented with 0.5% FOS and GOS [35], 0.15% MOS [36], 1% inulin [38], and also 1% resistant starch [40]. In cats, faecal butyrate and propionate concentrations exhibited a positive correlation with an increased abundance of *Lactobacillus* species when the animals were fed 0.4% XOS [37].

The onset and progression of disease states typically influence the production of SCFAs. For example, several investigations have indicated that canines and felines afflicted with inflammatory bowel disease (IBD) frequently exhibit gut microbiota dysbiosis, leading to a downregulation of SCFA-producing bacteria (e.g., *Bifidobacterium*, *Roseburia*) and a subsequent reduction in the synthesis of specific SCFAs [41,42,43]. However, given the heterogeneity observed across different studies, e.g., instances of elevated butyrate or propionate concentrations, further in-depth investigation is warranted.

### 3.2. Secondary Bile Acids

Primary bile acids, such as cholic and chenodeoxycholic acid, are lipid molecules produced in the liver and released into the digestive system through the biliary apparatus [44]. The gut microbiota transforms these host-derived bile acids into secondary bile acids [45], which constitute the majority of bile acids found in faeces and are prominent gut metabolites in dogs and cats [46]. A fundamental role of bile acids is to facilitate the digestion and absorption of fats [47]. Beyond their digestive function, bile acids act as signalling molecules, influencing glucose and lipid metabolism, insulin sensitivity, inflammatory responses, immune function, and thyroid-regulated energy expenditure [44,45,46,47,48].

A high-protein, high-fat, low-fibre diet (46.7% protein, 33.1% fat, 3.4% fibre, dry matter basis) composed primarily of minced beef (75%) has been demonstrated to elevate faecal deoxycholic acid levels in dogs [49]. Nevertheless, recent studies examining microbe-derived (secondary) bile acids in healthy dogs have yielded limited evidence regarding the impact of other dietary types. No statistical differences have been observed in studies comparing bone and raw food diets, grain-free diets, and high-protein, high-fibre diets to commercially available dry dog foods [50,51,52,53].

Cats fed a dry diet (33% protein, 7% fat, 9.3% fibre, dry matter basis) had a threefold decrease in total faecal bile acids compared to cats fed a wet diet (46% protein, 27% fat, 4.7% fibre) [54]. This finding implies that higher-fat and lower-fibre diets can induce an increase in total faecal bile acids in healthy cats. A more recent study evaluating 23 faecal bile acids in cats revealed significant alterations in bile acid profiles when comparing a 7.9% highly digestion-resistant starch diet (37.6% protein, 18.9% fat, 3.2% fibre) to a 0.5% low digestion-resistant starch diet (38.0% protein, 19.0% fat, 3.1% fibre) [55]. Specifically, 12 bile acids exhibited significant increases, while 3 showed reductions. The most pronounced changes were observed in the primary bile acids cholate and taurocholenate sulphate and the secondary bile acids 12-dehydrocholate and 3-dehydrocholate [55]. Therefore, despite similar macronutrient content, food processing techniques have a remarkable influence on microbial (secondary) bile acid metabolism [54,55]. In elder cats, a senior dry diet (31.1% protein, 16.8% fat, 1.3% fibre) containing particular ingredients (i.e., oat groats, broccoli, and tomato pomace) that protect against age-associated cognitive decline was formulated and compared to a commercial dry diet (32.7% protein, 19.1% fat, 1.4% fibre) in a crossover study [56]. The senior dry diet was associated with a significant decrease in the faecal secondary bile salts 7-alpha-hydroxy-cholestenone, dehydrolithocholate, deoxycholate, isoursodeoxycholate, lithocholate, and ursodeoxycholate and an increase in the faecal primary bile salt cholate [56].

In general, diet types and nutrient composition influence the canine and feline gut microbiota, and thus subsequentially alter the faecal bile acid pools. Based on the limited literature on dogs and cats, specific microbiome alterations induced by diet are impossible to predict. Conversely, several investigations have found alterations in secondary bile acid profiles in dogs and cats with various disease. Specifically, lower concentrations of faecal lithocholic acid, deoxycholic acid, and total secondary bile acids have been observed in dogs with chronic enteropathy and exocrine pancreatic insufficiency [57], and faecal bile acid dysmetabolism and reduced ursodeoxycholic acid correlated with novel microbial signatures in feline chronic kidney disease [58]. Further research is warranted to elucidate the relationship between these alterations and dietary factors, as well as the gut microbiota.

### 3.3. BCFAs and Indole Derivatives

The gut microbiome can alter the metabolism of amino acids, producing metabolites like branched-chain fatty acids (BCFAs) and indole derivatives [59]. These compounds influence various physiological processes, including immune function and inflammation [60].

The presence of BCFAs in the gut significantly impacts its environment, influencing the composition and activity of the gut microbiome [59]. In addition, these fatty acids act as signalling molecules, communicating with the gut lining and immune cells, ultimately helping to maintain gut barrier integrity, modulate immune responses and inflammation, and connect to cognitive function and mental health [59,60]. However, due to their carnivorous nature, dogs and cats have a higher dietary protein requirement and a lower level of colonic protein fermentation compared to humans and other omnivorous mammals [20,32]. Concentrations of BCFAs are thereby much smaller than SCFAs (i.e., 5% to 10% of SCFAs) [61], and are less extensively studied in current canine and feline nutrition research. The most important and abundant BCFAs in dogs and cats are isobutyrate and isovalerate, which are produced from valine and leucine, respectively. It is not surprising that BCFA production is directly related to crude protein content and amino acid concentrations in the diet [62]. However, research has observed that BCFA concentrations, particularly isobutyrate, decreased when dogs and cats were supplemented with dietary fibres (e.g., FOS, inulin) [63,64]. This indicates the colonic environment and microbiome function have shifted from protein fermentation to fibre fermentation. Similarly, GI disorders may precipitate a decline in BCFA production, as evidenced by diminished levels of isobutyrate and isovalerate in felines diagnosed with chronic enteropathy [43], an outcome plausibly linked to underlying microbiota dysbiosis.

Another significant group of amino acid metabolites comprises indole derivatives [23]. These compounds, originating from the amino acid tryptophan, function as signalling molecules and are crucial for immunomodulation (e.g., T-cell response), anti-inflammatory processes, and the maintenance of barrier functions within the gut [65,66]. Nonetheless, the health implications of various indole derivatives are inconsistent and markedly dose-dependent [67]. Excessive accumulation of certain compounds within the gut (e.g., indole sulphates) can lead to adverse effects, such as IBD and kidney dysfunction [67]. Similarly to BCFAs, indole derivatives (e.g., indole, indolin-2-one, indole-acrylate, indole-3-acetate) were increased with higher protein intake [20,32] and decreased or tended to be decreased by fibre supplementation in healthy dogs [68,69], but the interaction between diets and indole derivatives is rather complex. In a recent study comparing the effects of different protein levels (19.99%, 25.34%, 45.77%, dry matter basis [DM]) on gut health in dogs, faecal levels of indole and indolin-2-one significantly increased with protein consumption, while levels of indole-lactate, indole-acetate, indole-propionate, and 2-oxindole-3-acetate significantly decreased with increasing levels of protein consumed [62]. Tryptophan, which is the precursor amino acid of indoles, increased with decreased consumption of protein [62]. Nevertheless, none of these studies could clearly elucidate the dynamic changes in indole derivatives with dietary interventions and their specific role in gut health and host metabolism. In line with canine studies, faecal indole levels were decreased with the supplementation of dietary fibres (e.g., FOS, fructan) [70,71], but results concerning macronutrient levels exhibit an inconsistency, remaining unaffected in healthy cats [58,72].

## 4. Analytical Approach—Metabolomics

Metabolomics, encompassing the qualitative and quantitative analysis of all metabolites within biological systems, offers a powerful tool for elucidating the role of gut metabolites in both health and disease [20,21]. Utilizing distinct approaches, including targeted analysis, metabolic profiling, and metabolic fingerprinting [73], metabolomics allows for the measurement and identification of metabolites and the detection of changes in metabolite levels correlated with specific health conditions or dietary interventions [74]. Targeted analysis focusing on the precise quantification of predefined metabolites necessitates meticulous optimisation of extraction, separation, and detection, albeit with limited scope for identifying novel markers or broader metabolic shifts [75,76,77]. Metabolic profiling expands upon this, semi-quantitatively assessing a wider range of metabolites, typically within specific pathways, but remains less comprehensive [77,78]. Conversely, metabolic fingerprinting adopts a holistic, high-throughput approach to capture the overall metabolic state, foregoing individual metabolite identification in favour of rapid sample categorisation and pattern recognition for biomarker discovery [74,75,79,80]. Its capacity for large-scale comparative studies renders it particularly valuable [73].

A common metabolomic workflow involves the following subsequent stages: acquisition and preservation of samples (such as tissues and biofluids), sample processing, analysis and data capture, pre-processing and annotation of data, identification of metabolites, and biological interpretation [81]. Experimentation and optimisation are often necessary for sample preservation and processing to achieve the best results, particularly the choice of buffers (e.g., 80% methanol–20% water with 0.1% formic acid for polar metabolites by liquid chromatography–mass spectrometry), which are highly variable depending on the sample type, analytical platform, and metabolite properties [74,76].

The two most frequently employed analytical methods are NMR (nuclear magnetic resonance) spectroscopy and MS (mass spectrometry), which are typically coupled with separation procedures like LC (liquid chromatography) or GC (gas chromatography) [82] (Table 2). Other methods may include capillary electrophoresis (CE) and mass spectrometry imaging (MSI), but they are infrequently considered and have never been used in dogs or cats [83,84]. NMR is an unbiased, quick, uncomplicated, highly consistent, non-destructive and non-invasive technique that offers in-depth information on molecular structures in solution [13]. NMR has proved to be a powerful tool for analyte identification and quantification with little or no sample preparation and no prior chromatographic separation [85]. Conversely, UHPLC (ultra-high-performance liquid chromatography) joined with HRMS (high-resolution/high-mass-accuracy mass spectrometry) facilitates efficient separation and heightened sensitivity, specificity, and resolution, with exceptional mass accuracy below 3 ppm [81]. This is the reason that UHPLC-HRMS has become the primary analytical method in metabolomic research [81].

Data analyses of metabolomics generally involve the use of multiple software packages for raw data processing (e.g., MZmine (mzio GmbH, Kiel, Gemany), Sieve™ (Thermo Fisher Scientific, Waltham, MA, USA)), statistical analysis (e.g., SIMCA (Sartorius, Göttingen, Germany), MetaboAnalyst (McGill University, Montreal, QC, Canada)), metabolite identification (e.g., Human Metabolome Database (HMDB, TMIC, Edmonton, AB, Canada), Kyoto Encyclopaedia of Genes and Genomes (KEGG, Kanehisa Lab, Tokyo, Japan)), and pathway analysis and visualisation (e.g., MetaboAnalyst 6.0, Cytoscape 3.10 (Institute for Systems Biology, Seattle, WA, USA)) [74,75,76,77,78,79,80]. These tools cover various aspects of the analysis pipeline, which are also highly variable depending on the analytical platform and research goals.

Metabolomics can offer comprehensive insights into biofluids or tissue samples and aid in the detection of homeostatic imbalances within biological systems [85]. Most investigations in canine and feline metabolomics focus on plasma, serum, faeces, and urine, because they are non-invasive and more easily accessible [13]. Specifically, a range of substances found in faeces relate to various biological functions, such as nutrient digestion and absorption by the GI tract and microbiota [86]. Faecal metabolomics can and is mostly used to study the GI metabolome and host–microbe interactions, as well as to identify disease biomarkers such as GI dysbiosis [13].

## 5. Dietary Intervention on the GI Metabolome

Investigation of the gut metabolome by means of faecal metabolomics is still relatively new [13,87]. Very few studies have investigated the gut metabolome in dogs and cats specifically, with the vast majority focusing on the identification of potential markers for diseases, such as IBD and kidney disease [9,26,88]. It has been demonstrated, however, that nutritional intervention is able to induce changes in the canine and feline metabolome, the currently available research on which is discussed below and summarised in Table 3.

### 5.1. Nutrient Composition, Diet Types, and Ingredients Used

Human studies have indicated that increased dietary protein levels can facilitate weight loss, but may potentially induce gut dysbiosis [89]. To evaluate whether these results also apply to dogs, there is a need for more studies on microbiome and metabolome changes in dogs fed high-protein diets. One study demonstrated a potential negative health effect: Ephraim et al. [62] observed that a high-protein diet (42%) significantly increased faecal levels of putrefaction-associated metabolites, including tryptophan, indole, and indolin-2-one, and inflammation-associated metabolites such as 12,13-DiHOME and 9,10-DiHOME, but decreased several beneficial indoles, including indole-propionate, indole-acetate, and 2-oxindole-3-acetate, which are important to maintain gut barrier integrity [62]. These findings are likely linked to gut inflammation, which implies that the high protein intake may induce relevant health risks in dogs [62]. Despite the successful weight loss with the high-protein diet, metabolomic changes did not demonstrate a consistent effect in obese dogs. For instance, in a weight loss program for obese dogs, a high-protein high-fibre diet (37% protein, 18% fibre, DM) significantly changed 13 metabolites, including increased epicatechin, matairesinol, 1,5-isoquinolinediol, and 2-hydroxiquinoline, and decreased purine, L-methionine, coumestrol, and the alkaloids 1-methylxanthine and trigonelline [90]. Nevertheless, these results do not allow us to draw firm conclusions, as they contradict findings of other weight loss dog/human and/or other high-protein high-fibre studies, which often showed increased putrefaction (e.g., NH_3_, indole) and altered SCFAs [62,69,91]. Similarly, a recent study revealed distinct metabolomic profiles in dogs fed a high-protein low-carbohydrate (50% protein, 32% carbohydrate, DM) vs. a low-protein high-carbohydrate (18% protein, 62% carbohydrate, DM) diet, whereas no potential adverse health effects were observed [20]. Conversely, this study surprisingly demonstrated that the high-protein low-carbohydrate diet significantly influenced gut functions of amino acid and lipid metabolism and vitamin biosynthesis, which are potentially associated with promoting weight loss and immune function [20]. In general, these studies highlight a disparity in the observed health effects of high-protein diets between human and canine research. Further investigations are therefore urgently needed.

Beyond the focus on protein, the current metabolomic research in canines and felines rarely delves into the sources or compositions of other essential nutrients. To the authors’ knowledge, only one study has revealed the metabolomic changes in dogs fed a high-fat vs. a high-starch diet. Nevertheless, only ten annotated metabolites (i.e., methionine and nine di-/tri-peptides) were putatively identified as marker molecules of the high-starch diet, high-fat diets, with five unknown metabolites upregulated [14]. In a study focused on testing the health effects of new ingredients in dog food, adding 25% *w*/*w* cooked navy beans induced higher faecal levels of 2-piperidinecarboxylic acid, s-methyl-cysteine, -sitosterol, -tocopherol, sucrose, and fructose compared to the commercial control diet [92]. These changes were linked to the regulation of lipid and carbohydrate metabolism, suggesting cooked navy beans could be used as a new functional ingredient to improve gut function [92].

Diet types and ingredients used influence the metabolome, which has also been demonstrated in dogs and cats. The comparison between dry and wet diets remains a subject of considerable interest in canine and feline nutrition [88]. Recent research has reported higher levels of butyric acid in cats fed dry food compared to those fed canned food [93]. Interestingly, the act of softening dry food with water has been shown to elicit altered metabolomic profiles, with 24 marker metabolites and pathways associated with purine (nucleotide) and riboflavin (cofactor and vitamin) metabolism being significantly affected [94]. The differences between a BARF (bones and raw food) and commercial diet, for example, were assessed in 46 dogs using untargeted metabolomics [47]. Increased cholesterol, 4-hydroxybutryric acid, and 4-aminobutyric acid were observed in the faecal samples of the BARF group, indicating a shift in the gut metabolome due to different diet types and ingredients used [47]. Liao et al. [95] conducted a study comparing the faecal microbiome and metabolic profiles in healthy puppies undergoing a dietary transition from a salmon-based to a poultry-based dry diet. They observed that eight compounds, i.e., L-asparagine, L-histidine, thiamine, carnosine, histamine, citric acid, spermine, and 5-aminolevulinic acid, were significantly upregulated. Conversely, seven compounds, i.e., indoleacetic acid, serotonin, 5-hydroxyindoleacetic acid, corticosterone, phenylacetic acid, 4-pyridoxic acid, and choline, were significantly downregulated in dogs experiencing an abrupt dietary change compared to those undergoing a gradual transition [95]. These marker metabolites are likely associated with amino acid and lipid metabolism [95]. Investigations have shown that the larvae of the black soldier fly (*Hermetia illucens*) have considerable utility for incorporation into pet food products [96,97]. A recent study indicated that a 20% substitution of chicken meal with defatted black soldier fly larvae protein in a dry diet led to significant shifts in the faecal metabolome, with 54 marker metabolites displaying pronounced changes [98]. These metabolic changes were observed across 18 pathways involved in arachidonic acid metabolism, arginine biosynthesis, and the interconversion of pentose and glucuronate compounds [98].

### 5.2. Dietary Supplements

Despite the limited number of studies, current research on canine and feline gut metabolomics also includes investigation of the effects of dietary supplements, such as dietary fibre and pre-, pro-, and synbiotics.

It has been demonstrated that dietary supplementation of fibre and prebiotics has a beneficial impact on the gut metabolome [55,99]. In cats, a highly resistant starch (8% DM) diet significantly changed 344 faecal metabolites, particularly increased faecal butyrate, ketones, indole-lactate/indole-propionate, and polyamines, and decreased endocannabinoid N-acylethanolamines, implying an improvement in the gut environment and host health [55]. In a recent study of glucose metabolism in obese dogs, Apper et al. [99] observed that scFOS supplements resulted in marked alterations in the faecal metabolome, including increased SCFAs, bile acids, and several amino acids. These results indicate that scFOS supplementation modulate gut microbiota composition and activity, which benefitted glucose homeostasis in obese dogs [99].

Few studies have demonstrated the positive health effect of pro- and synbiotics in dogs and cats. Probiotic cheese containing *Lactobacillus reuteri* KACC 92293 and *Bifidobacterium longum* KACC 91563 was fed to healthy beagles to investigate its influence on the faecal metabolic profile, where 55 marker metabolites were identified [100]. Particularly, the probiotic supplement increased the levels of acetic, propionic, and 4-aminobutyric acids, implying positive changes in gut metabolites and the intestinal environment in dogs [100]. The previously mentioned study by Whittemore et al. [101] also evaluated the effect of a synbiotic on the faecal metabolome of antibiotic-administered cats, in which the symbiotic significantly altered 106 metabolites, including several SCFAs, bile acids, and polyamine pathway metabolites. Synbiotic supplements could thus aid in remedying the gut dysbiosis caused by antibiotics [101].

Gut metabolomic studies on dietary supplements other than those previously mentioned remain limited. The impact of gallic acid, however, has been evaluated in dogs by Yang et al. [102,103]. Their research indicated that long-term (45 days) supplementation with 0.08% gallic acid significantly affected faecal levels of 37 metabolites, such as succinic acid, L-asparagine, spermidine, ciliatine, and adenosine [98]. These metabolites are associated with nine metabolic pathways: carbohydrate (butanoate and propanoate metabolism, TCA cycle), amino acid (alanine, aspartate, and glutamate; arginine and proline; glutathione; beta-alanine; and phosphonate metabolism), and nucleotide metabolism (purine metabolism) [102]. Furthermore, dietary supplementation with 0.05% gallic acid was found to substantially alleviate transport stress and significantly regulate 48 faecal metabolites [103]. These metabolites are linked to several metabolic pathways, including amino acid metabolism (cysteine and methionine metabolism; tyrosine metabolism; valine, leucine, and isoleucine degradation; valine, leucine, and isoleucine biosynthesis; lysine degradation; and taurine and hypo-taurine metabolism), carbohydrate metabolism (glycolysis/gluconeogenesis, pyruvate metabolism, and fructose and mannose metabolism), lipid metabolism (glycerol-lipid metabolism, fatty acid biosynthesis, primary bile acid biosynthesis, and alpha-linolenic acid metabolism), and nucleotide metabolism (purine metabolism) [55]. Zhang et al. [104] evaluated the effect of dietary supplementation of methyl-sulphonyl-methane (MSM) and myo-inositol (MI) in poodles. A total of 149 metabolites were obtained by the supplementation that are associated with pathways of carbohydrate, nucleotide, amino acids, cofactors and vitamins, and lipid metabolism [104].

**Table 3:** Summary of typical research on the effects of nutritional intervention on the canine and feline gut metabolome.

Reference	Design	Type	Method	Significant Findings
[14] Lyu et al., 2022	High-starch (63.5%) vs. high-fat (46.9%) dog diets (*n* = 10)	Untargeted	UPLC-MS	High starch increased methionine and nine di/tripeptides
[20] Lyu et al., 2023	High- (50%) vs. low-protein (17.8%) dry dog diets (*n* = 12)	Targeted and untargeted	UPLC-MS	High protein: amino acid and lipid metabolism, vitamin biosynthesis Low protein: affected carbohydrate fermentation
[52] Schmidt et al. 2018	BARF (*n* = 27) vs. dry diet in dogs (*n* = 19)	Untargeted	GC-TOF–MS	Increased cholesterol, 4-hydroxybutryric acid, and 4-aminobutyric acid
[62] Ephraim et al., 2020	Dry diet with three protein levels (17%, 30%, 42%) in dogs (*n* = 10)	Untargeted	Unspecified	42%—increased indole, tryptophan, indolin-2-one, decreased indole-acetate, indole-propionate, 2-oxindole-3-acetate
[92] Forster et al. 2015	Dry diet adding cooked navy beans in dogs (*n* = 10)	Targeted and untargeted	GC–MS + UPLC–MS	Greater levels of 2-piperidinecarboxylic acid, *S*-methyl-cysteine, -sitosterol, -tocopherol, sucrose, and fructose in the navy bean diet
[93] Bian et al., 2023	Dry vs. wet food in cats (*n* = 9)	Untargeted	UPLC-MS	Dry: higher butyric acid
[94] Zhang et al., 2022	Dry vs. water-softened dry food in dogs (*n* = 10)	Targeted and untargeted	GC–MS + UPLC–MS	Water-softened dry food influenced the purine metabolism, riboflavin metabolism
[95] Liao et al., 2023	Abrupt (*n* = 7) vs. gradual dietary transition (*n* = 6) in dogs	Targeted and untargeted	GC–MS + UPLC–MS	Abrupt change: decreased L-asparagine, L-histidine, thiamine, carnosine, histamine, citric acid, spermine, 5-aminolevulinic acid; increased indoleacetic acid, serotonin, 5-hydroxyindoleacetic acid, phenylacetic acid, corticosterone, 4-pyridoxic acid, choline
[98] Jian et al., 2022	Control vs. black soldier fly-based dry diet in dogs (*n* = 7)	Targeted and untargeted	GC–MS + UPLC–MS	Decreased propionate, butyrate, SCFAs, isobutyrate, isovalerate, and BCFAs; affected arachidonic acid metabolism, arginine biosynthesis, pentose and glucuronate interconversions
[55] Jackson et al., 2020	Highly resistant starch (8%) dry diet in cats (*n* = 6)	Untargeted	GC–MS + UPLC–MS	Increased butyrate, ketones, indole-lactate/indole-propionate, and polyamines, decreased endocannabinoid N-acylethanolamines
[99] Apper et al. 2020	Adding scFOS to obese dogs (*n* = 6)	Untargeted	GC–MS + UPLC–MS	Several bile acids and amino acids
[100] Kim et al., 2020	Dogs fed probiotics *L. reuteri* and *B. longum* (*n* = 4)	Untargeted	GC–MS + UPLC–MS	Increased acetic, propionic, and 4-amino- butyric acids
[101] Whittemore et al., 2019	A synbiotic in cats fed antibiotics (*n* = 8)	Untargeted	GC-TOF–MS	SCFAs, bile acid, tryptophan, sphingolipid, polyamine, benzoic acid, and cinnamic acid pathways
[102] Yang et al., 2022	Dry dog diet with 0.02, 0.04, 0.08% gallic acid (*n* = 5)	Targeted and untargeted	UPLC–MS	0.08%—affected 37 metabolites including SCFAs, succinic acid, L-asparagine, spermidine, ciliatine, and adenosine; and pathways of carbohydrate, amino acid, and nucleotide metabolism
[103] Yang et al., 2022	0.05% Gallic acid in puppies with transport stress (*n* = 6)	Untargeted	UPLC-MS	Gallic acid affected 48 metabolites involved in amino acid, carbohydrate, lipid, and nucleotide metabolism pathways
[104] Zhang et al., 2024	Dry diet with 0.2% MSM and/or MI added in dogs (*n* = 8).	Targeted and untargeted	GC–MS + UPLC–MS	MSM and MI affected carbohydrate, amino acid, lipid, cofactor, vitamin, and nucleotide metabolism

BARF, bones and raw food; GC, gas chromatography; TOF, time of flight; MS, mass spectrometry; UPLC, ultra-high-performance liquid chromatography; scFOS, short-chain fructo-oligosaccharide; SCFA, short-chain fatty acid; BCFA, branched-chain fatty acid; MSM, methyl-sulphonyl-methane; MI, myo-inositol.

## 6. Conclusions, Limitations, and Perspectives

The gut metabolome plays a vital role in pet nutrition, influencing digestion, immune function, and overall health [47]. As research advances, a deeper understanding of the gut metabolome will lead to more effective and personalised pet nutrition practices [14]. However, several significant limitations have emerged in current pet nutrition research, presenting challenges and necessitating a shift in focus for future investigations.

In addition to the relatively limited research on the gut metabolome compared to human studies, a major limitation in current canine and feline metabolomic research lies in the absence of combined metabolome and microbiome analysis. The integration of these two approaches can offer comprehensive insight into the relationship between dietary intake, the gut microbiota, and host metabolism [20,42]. Moreover, it may enable the identification of certain microbial metabolites associated with changes in host metabolism and overall health, as well as provide a better understanding of the mechanisms involved in the impact of dietary interventions on the gut microbiota and host metabolism [5]. Nevertheless, almost all studies on the canine and feline gut metabolome dwell on characterizing metabolomic alterations induced by dietary interventions, with limited exploration of the complex interplay among the gut microbiota, host metabolism, and dietary influences [20]. Existing technical approaches offer viable means by which future research can achieve this integration. These include metagenomic sequencing and meta-transcriptomics, which provide comprehensive data on metabolic pathways and processes operating within the microbial community [105]; correlation and network analyses, enabling the assessment and visualisation of relationships between microbes and metabolites [106]; and pathway analysis, which facilitates the identification of affected metabolic pathways [106].

Furthermore, the majority of canine and feline studies examining metabolites primarily focus on overall abundance levels, neglecting a more in-depth understanding of specific marker metabolites. For instance, the modulation of certain biogenic amines (e.g., 5-hydroxytryptamine) and bile acids (e.g., ursodeoxycholic acid) through dietary intervention has been explored for targeted nutritional and/or therapeutic purposes in human research [107,108], yet remains largely uninvestigated in dogs and cats. This limits our ability to pinpoint the precise metabolic pathways (e.g., tryptophan metabolic pathway) and biological processes (e.g., the gut microbiome-bile acid axis) that are being altered by dietary interventions or other factors. Future research should prioritise the elucidation of alteration patterns, regulatory mechanisms, and clinical implications pertaining to the aforementioned (and other) metabolites involved in key metabolic pathways and physiological processes. It remains challenging to draw definitive conclusions about the significance of the differential metabolites, since the total number of the gut metabolites is quite large, and it is still unclear whether their roles are primary or secondary [4]. While the combination of untargeted and targeted metabolomics could potentially address this limitation, the identification and functional understanding of many metabolites remain unexplored even in humans, further complicating the interpretation of findings in dogs and cats [14,20]. As such, the conclusions drawn from current research may be incomplete. Additionally, the exact links among food, microbiota, host, and their interaction are still unclear, and it remains controversial whether altered microbiota and their metabolites are the cause, consequence, or epiphenomena [5]. Therefore, it is necessary to update the discussion and conclusions of current studies once new relevant results or insights emerge in the future.

Species difference between humans and pets is another major limitation. Since metabolomics is emerging in canine and feline nutrition, detailed information about the identification and functionality of many metabolites is often extrapolated from humans or other mammals [14]. This may lead to misunderstandings or deviations between the experimental conclusions and the actual reality. For instance, marked discrepancies in the observed adverse health effects of high-protein diets have been noted between human and canine populations [62,90,91]. This disparity is likely attributed to the carnivorous nature of dogs, suggesting a potentially higher tolerance to the adverse effects of high protein intake compared to humans [20,47]. Therefore, once new species-specific knowledge and/or databases become available in the future, study interpretations must be updated.

Other limitations in current canine and feline metabolomic studies may include insufficient details on food ingredients (e.g., fatty acid profiles of fat sources, amino acid profiles of protein sources), variability in technical specifications (e.g., UHPLC vs. NMR; lipidomics), and suboptimal experimental designs (e.g., sample sizes, dog species, time/dose effects). However, these limitations are expected to be addressed by the emergence of more diverse and in-depth studies in the near future, leading to a more complete understanding of canine and feline metabolomes.

## Figures and Tables

**Table 1:** Overview of typical metabolites generated by host endogenous microbiota in the gut of dogs and cats.

Metabolic Substrates	Representative Metabolites
Carbohydrates	SCFAs, BCFAs, gases (CO_2_ and CH_4_), oligomers (disaccharides, oligosaccharides), organic acids (succinate, lactate, pyruvate), ethanol, methanol
Proteins	SCFAs, BCFAs, biogenic amines, amino acids, phenols, p-cresols, indoles, gases (CO_2_, H_2_S, NH_4_, CH_4_)
Lipids	conjugated fatty acids, acylglycerols, sphingomyelin, phosphatidylcholines, cholesterol, triglyceride, phosphor-ethanol-amines
Bile acids	cholate, hyocholate, deoxycholate, chenodeoxycholate, α-muri-cholate, β-muri-cholate, ω-muri-cholate, taurocholate
Choline metabolites	methylamine, dimethylamine, trimethylamine, trimethylamine N-oxide
Bacterial cell wall	lipopolysaccharide A, peptidoglycan, sphingomyelin, phosphor-ethanol-amines
Vitamins	vitamin K, vitamin B12, biotin, folate, thiamine, riboflavin, pyridoxine
Polyphenols	hydroxyphenyl-propionic acid, pyrocatechol, enterodiol
Polyamines	putrescine, cadaverine, spermidine
Volatile compounds	aldehydes, ketones, alkanes, benzenoids, organosulphur
Endocannabinoids	N-palmitoyl-ethanol-amine, 2-arachidonoylglycerol, N-oleoyl-ethanolamine
N-acetyl compounds	N-acetyl-tryptophan, N-acetyl cysteine, N-acetyl glucosamine
Others	γ-aminobutyric acid, exopolysaccharides, phenolic, benzoyl, hormones, phenyl derivatives

**Table 2:** Comparison of different metabolomic methods.

Approach	Advantages	Limitations	Costs
NMR	High throughput Non-destructive Highly reproducible Provides detailed information on structure and dynamics of molecules Sensitive to small changes	Relatively lower sensitivity Limited number of metabolites detectable Requires specialised expertise	Expensive
GC-MS	Excellent sensitivity and resolution High throughput Wide range of metabolites detectable Can be used for quantitative analysis	Limited to volatile metabolites Requires extensive sample preparation	Average
LC-MS	High sensitivity and resolution Wide range of metabolites detectable Flexible for different sample types Can be used for quantitative analysis	Time-consuming Requires high-quality standards Complex sample preparation Requires specialised expertise	Average
MSI	Provides spatial information about metabolite distribution Can be used for studying tissue heterogeneity	Limited to specific types of samples Difficult to interpret data	Very expensive
CE	Excellent resolution High sensitivity Can be used for quantitative analysis	Limited to small molecules Time-consuming	Low cost

NMR, nuclear magnetic resonance; GC, gas chromatography; MS, mass spectrometry; LC, liquid chromatography; CE, capillary electrophoresis; MSI, mass spectrometry imaging.

## Data Availability

Not applicable.

## Abbreviations

The following abbreviations are used in this manuscript:

GI	gastrointestinal
SCFAs	short-chain fatty acids
BCAAs	branched-chain amino acids
BCFAs	branched-chain fatty acids
FOSs	fructo-oligosaccharides
GOSs	galacto-oligosaccharides
MOSs	manno-oligosaccharides
XOSs	xylo-oligosaccharides
DM	dry matter
NMR	nuclear magnetic resonance
MS	mass spectrometry
LC	liquid chromatography
GC	gas chromatography
CE	capillary electrophoresis
MSI	mass spectrometry imaging
UPLC	ultra-high-performance liquid chromatography
HRMS	high-resolution/high-mass-accuracy mass spectrometry
BARF	bones and raw food
TOF	time of flight
MSM	methyl-sulphonyl-methane
MI	myo-inositol
